# UV‐C‐induced reactive carbonyl species are better detoxified in the halophytic plants *Salicornia brachiata* and *Arthrocnemum macrostachyum* than in the halophytic *Sarcocornia fruticosa* plants

**DOI:** 10.1111/tpj.70239

**Published:** 2025-05-27

**Authors:** Jaykumar Patel, Kusum Khatri, Tesfaye Asmare Sisay, Zai Du Nja, Babita Choudhary, Zhadyrassyn Nurbekova, Anmol Mishra, Noga Sikron, Dominic Standing, Anurag Mudgal, Varsha Mudgal, Moshe Sagi

**Affiliations:** ^1^ Jacob Blaustein Center for Scientific Cooperation, The Jacob Blaustein Institutes for Desert Research Ben‐Gurion University of the Negev Sede Boqer Campus Sede Boker 8499000 Israel; ^2^ The Albert Katz International School for Desert Studies, The Jacob Blaustein Institutes for Desert Research Ben‐Gurion University of the Negev Sede Boqer Campus Beer Sheva 8499000 Israel; ^3^ The Albert Katz Department of Dryland Biotechnologies, French Associates Institute for Agriculture and Biotechnology of Dryland, The Jacob Blaustein Institutes for Desert Research Ben‐Gurion University of the Negev Sede Boqer Campus Beer Sheva 8499000 Israel; ^4^ Department of Mechanical Engineering, School of Technology Pandit Deendayal Energy University Gandhinagar 382426 India; ^5^ Katif Research Center Sedot Negev Israel; ^6^ Ministry of Science and Technology Netivot Israel

**Keywords:** aldehyde oxidase (AO), *Arthrocnemum macrostachyum*, halophyte, reactive carbonyl species (RCS), *Salicornia brachiata*, UV‐C irradiation

## Abstract

Abiotic stress‐induced reactive carbonyl species (RCS) accumulation in plants stimulates oxidative stress by DNA adduct formation, protein carbonylation, and antioxidant pool depletion, triggering senescence or programmed cell death. RCS accumulation under abiotic stress has rarely been studied in halophytic plants that are adapted to highly saline environments. In the current study, exposure to UV‐C irradiation resulted in a higher RCS accumulation in the halophytic *Sarcocornia fruticosa* ecotypes VM and EL than in *Salicornia brachiata* (SB) and *Arthrocnemum macrostachyum* (AM). Accordingly, SB and AM recovered better, whereas VM and EL showed significant damage 14 days after UV‐C application. Reduced aldehyde oxidase (AO) activity, recently shown to detoxify carbonyl aldehydes in *Arabidopsis* plants, is likely responsible for the significantly higher RCS accumulation and damage in the VM and EL plants. As evidence for this, the VM plants exposed to exogenously applied 3 mM of malondialdehyde or 3 mM of benzaldehyde exhibited decreased AO activity, which resulted in the accumulation of endogenous RCS and severe damage, including mortality. In contrast, the AM plants were able to detoxify RCS by AO activity enhancement, exhibiting recovery after 25 days. These results highlight the role of RCS accumulation in VM and EL plant tissue damage, while improved AO activity, which resulted in improved RCS detoxification in SB and AM, promoted better recovery.

## Significance Statement

UV‐C irradiation induces tissue damage, enhancing reactive carbonyl species in *Sarcocornia fruticosa* halophytes but not in *Salicornia brachiata* and *Arthrocnemum macrostachyum*. The higher AO activity induced by UV‐C irradiation is the cause of better resistance to stress in *Salicornia brachiata* and *Arthrocnemum macrostachyum*.

## INTRODUCTION

Abiotic stresses, such as UV‐C irradiation, salinity, heavy metals, and others, have been shown to cause an increase in reactive carbonyl species (RCS) accumulation in plants (Liang et al., 2023; Nurbekova et al., 2021; Sultana, Sakurai, Biswas, Szabados, & Mano, 2024). This increase is mainly due to two reasons: (1) Excess reactive oxygen species (ROS) react with the polyunsaturated fatty acids present in cellular membranes, producing lipid hydroperoxides (Biswas & Mano, 2015), and (2) abiotic stress‐induced lipoxygenases (LOXs) also generate lipid hydroperoxides (Chen, 2021). These lipid hydroperoxides contribute to reductive fission in plant cells, leading to RCS generation, including reactive aldehydes and ketones (Matamoros et al., 2018).

RCS are integral parts of the plant's cellular metabolism, constitutively generated either as intermediate metabolites or as a final product. Abscisic aldehyde and indole acetaldehyde are examples of intermediate RCS produced during the biosynthesis of the phytohormones abscisic acid (ABA) and indole acetic acid (IAA), respectively (Koshiba, Saito, Ono, Yamamoto, & SatÔ, 1996; Seo et al., 1998; Seo et al., 2004). Plants also produce some aromatic and aliphatic RCS as a final product, such as benzaldehyde, cinnamaldehyde, phenylacetaldehyde, and n‐hexanal. These RCS were identified as involved in flavor and aroma production, flower scent production, wounding/herbivory response, plant communication, and defense (Chehab et al., 2008; Gutensohn et al., 2011; Huang, Li, Fu, & Dudareva, 2022; Schwab, Davidovich‐Rikanati, & Lewinsohn, 2008; Verma et al., 2017).

Keeping the RCS concentration in balance is essential for efficient plant metabolism. Increased RCS concentrations can lead to (1) the formation of DNA adducts, (2) the irreversible inactivation and carbonylation of proteins, and (3) the consumption of reduced glutathione from cells (Biswas & Mano, 2021; Jaballi & Missihoun, 2022; Mano, Miyatake, Hiraoka, & Tamoi, 2009). Rapid loss of glutathione increases the redox potential and depletes the ascorbate pool, accelerating oxidative stress and creating a vicious cycle of RCS and ROS, inactivating various target proteins and ultimately leading to senescence or programmed cell death [PCD (Mano, 2012)].

Increased concentrations of RCS such as acrolein and malondialdehyde (MDA), in response to stress conditions, irreversibly modify chloroplast‐localized Rubisco and oxygen‐evolving complex (OEC33) proteins, leading to a decrease in photosynthesis in *Arabidopsis* leaves (Mano et al., 2009; Yamauchi et al., 2008; Yamauchi & Sugimoto, 2010). Among RCS, α,β‐unsaturated RCS, such as acrolein, 4‐hydroxyl‐2‐nonenal (HNE), trans‐4‐hydroxy‐2‐hexenal (HHE), trans‐2‐hexenal, and trans‐2‐nonenal, were shown to be more toxic than saturated MDA, acetaldehyde, and methylglyoxal to the cells in *Spinacia oleracea* leaves (Mano et al., 2009). Abiotic stress‐induced RCS was shown to result in PCD in *Arabidopsis* root and tobacco BY2 cells (Biswas & Mano, 2015), and exogenously applied RCS, such as methylglyoxal, benzaldehyde, nonanal, *trans*‐2‐nonanal, and hexanal, resulted in reduced growth, damage symptoms, and early senescence in *Arabidopsis* seedlings and *Arabidopsis* plant leaves (Hoque, Uraji, Hoque, Nakamura, & Murata, 2017; Nurbekova et al., 2021, 2024). Significantly, the accumulation of RCS, rather than ROS, was suggested as the primary reason for damage in *Arabidopsis* plants exposed to salinity stress (Sultana et al., 2024). Accordingly, the application of exogenous *trans*‐2‐hexenal, together with aluminum stress, increased lipid hydroperoxide levels. It promoted PCD in wheat roots, while carnosine supplementation, the α,β‐unsaturated aldehyde scavenger, reduced the damage in wheat roots caused by the aluminum stress (Liang et al., 2023).

In plants, RCS are mostly detoxified by aldehyde dehydrogenase, aldo‐keto reductase, and 2‐alkenal reductase. Recently, it was demonstrated that among the four aldehyde oxidases (AOs) in *Arabidopsis*, AO3 in leaves and AO4 in siliques can detoxify RCS in *Arabidopsis* plants exposed to UV‐C irradiation (Nurbekova et al., 2021, 2024; Srivastava et al., 2017). While studies related to plant abiotic stress responses and RCS have been performed with glycophytic plants, such studies with halophytic plants are rare. In the current study, the edible halophytic plants *Sarcocornia fruticosa* [ecotypes VM and EL (Ventura & Sagi, 2013)], *Salicornia brachiata* [SB (Patel et al., 2024)], and *Arthrocnemum macrostachyum* [AM (Sisay et al., 2022)] were exposed to UV‐C irradiation, and their stress responses were investigated. SB and AM showed significantly fewer damage symptoms than VM and EL, resulting from lower RCS content in the shoot tissue. As evidence for this, MDA and benzaldehyde, exogenously applied to the VM and AM shoots, resulted in a significant endogenous RCS enhancement and severe damage in the VM shoots, whereas much less damage, in addition to the lack of significant increased RCS accumulation, was noticed in the AM shoots 25 days after the application. The significantly fewer damage symptoms in SB and AM are attributed to the substantially higher AO activity present in the SB and AM shoots.

## RESULTS

### VM and EL are more susceptible to UV‐C irradiation than SB and AM

**Figure 1:**
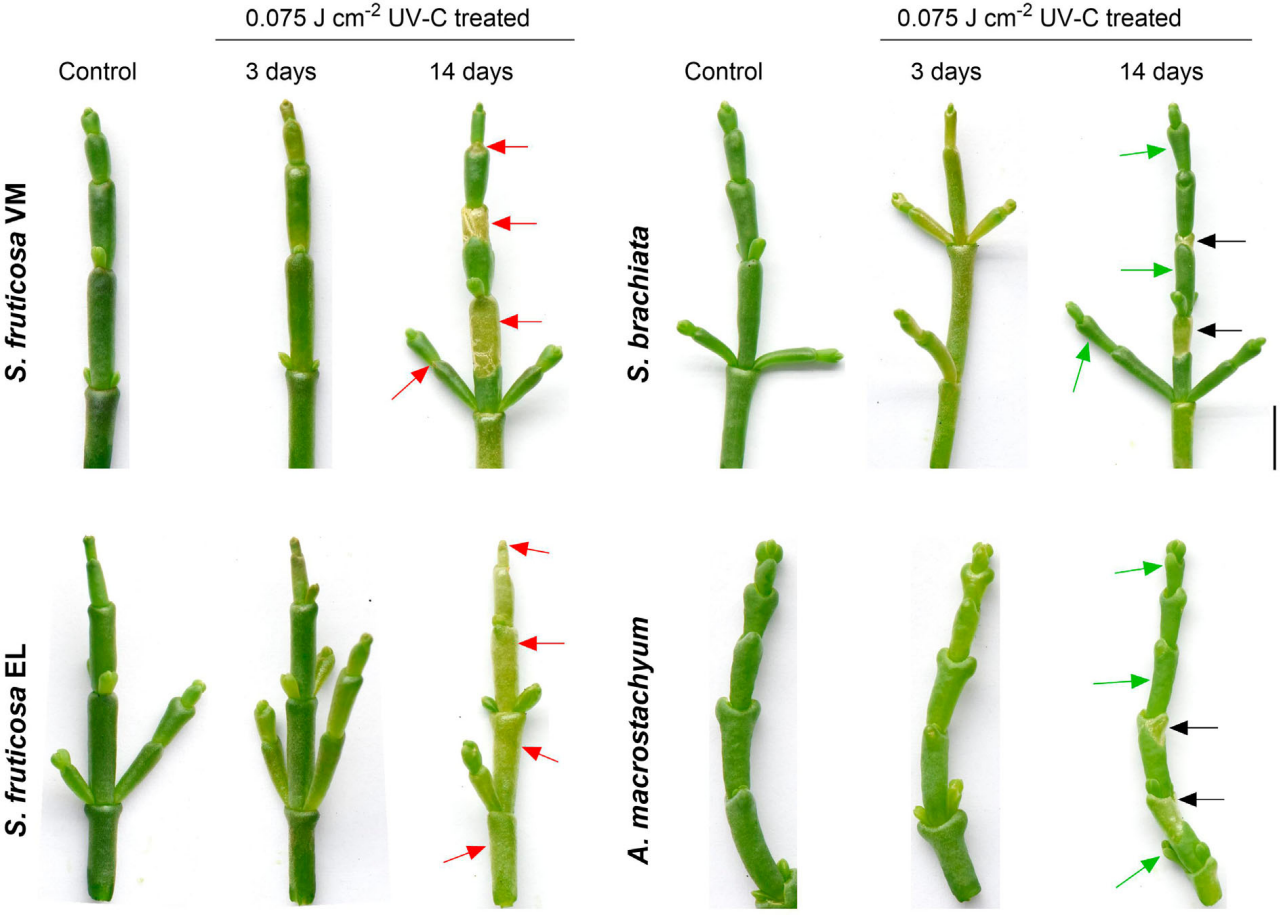
Effect of 0.075 J cm^−2^ UV‐C irradiation on the appearance of *Sarcocornia fruticosa* ecotypes VM and EL, *Salicornia brachiata* (SB), and *Arthrocnemum macrostachyum* (AM) halophyte plants. The top 5 cm of the main shoot tips were cut and imaged 3 and 14 days after UV‐C application. Red arrows indicate extensive damage caused by UV‐C on *S. fruticosa* ecotypes VM and EL. Black arrows indicate scars left behind on *S. brachiata* and *A. macrostachyum* plants during recovery after UV‐C application. Green arrows indicate new growth after recovery. The scale bar represents 1 cm.

Three days after exposure to UV‐C irradiation (0.075 J cm^−2^), examination of the halophytic plants showed surface damage on the shoot tips (including chlorosis, yellowing, blackened shoot tips, and dried outer cuticles) (Figure 1). However, 14 days after the UV‐C treatment, SB and AM showed new shoot tip growth and signs of post‐stress recovery. In contrast, the VM and EL plants did not recover, exhibiting more significant damage, including chlorosis, cracks, and roughness on the outer cuticle layer (Figure 1).

**Figure 2:**
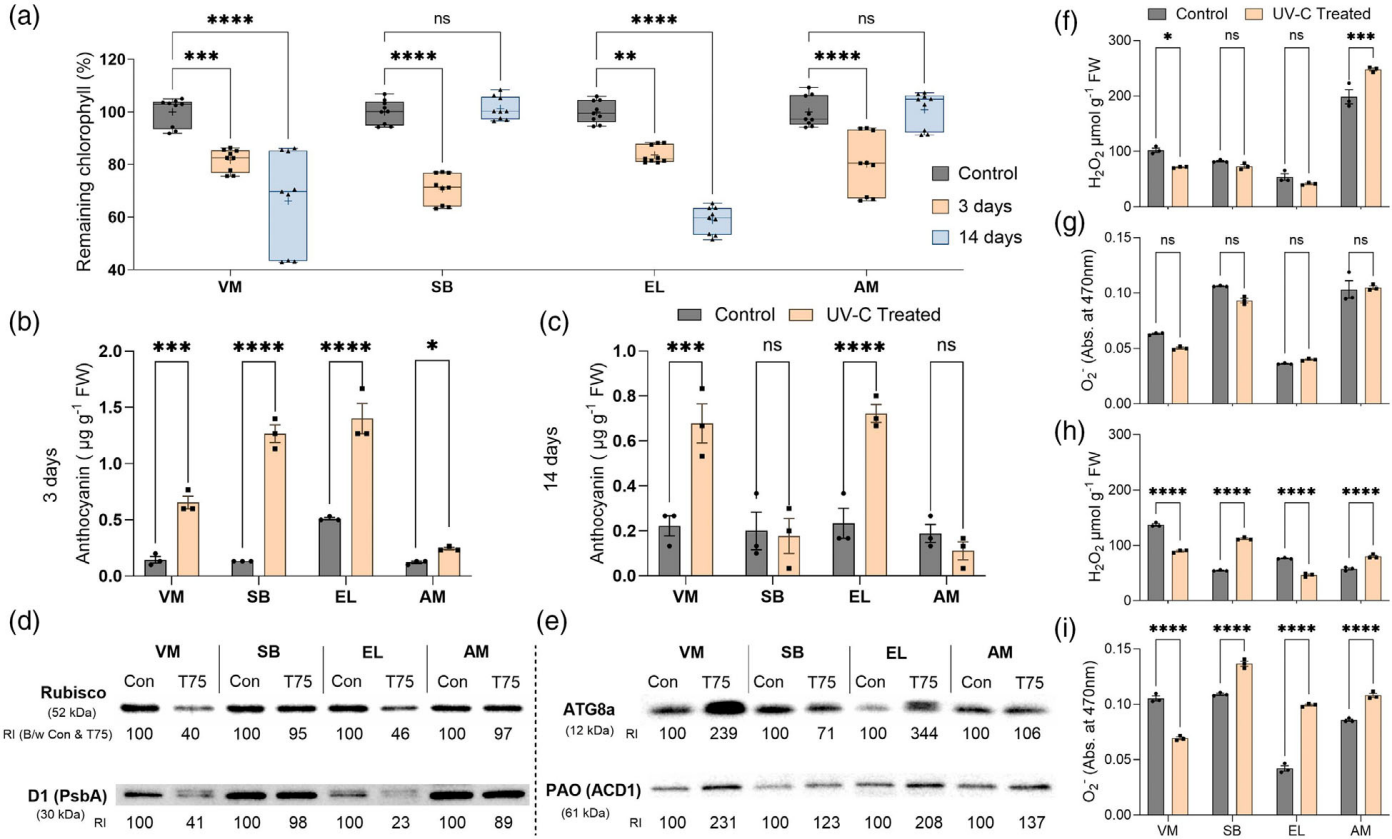
Effect of 0.075 J cm^−2^ UV‐C irradiation on the levels of remaining chlorophyll, anthocyanin, chloroplast‐localized proteins, H_2_O_2_, and O_2_^−^ in *Sarcocornia fruticosa* (ecotypes VM and EL), *Salicornia brachiata* (SB), and *Arthrocnemum macrostachyum* (AM). (a) Relative chlorophyll content 3 and 14 days after UV‐C treatment. Data represent three biological replicates from three independent experiments (*n* = 9). Box plot shows min to max values with 25th and 75th percentile boxes; lines in the box indicate the median, whereas plus signs indicate the mean value. (b) Anthocyanin accumulation 3 days after UV‐C stress. (c) Anthocyanin accumulation 14 days after UV‐C stress, *n* = 3, three samples from three independent experiments, and the error bar indicates standard error. (d) Western blotting of photosynthesis‐related proteins Rubisco (52 kDa) and D1 (PsbA) (30 kDa). (e) Western blotting of the senescence‐related proteins PaO (ACD1) (61 kDa) and ATG8a (12 kDa). Chemiluminescence values of the blots were recorded in the ChemiDoc Touch imaging system (Bio‐Rad Laboratories, USA). Image analyses and relative intensity calculations between the control and treated samples were performed using Image Lab (Bio‐Rad Laboratories, USA) and ImageJ software (https://imagej.nih.gov/ij/). “Con” represents protein samples from control plants, and “T75” represents samples from plants treated with 0.075 J cm^−2^ UV‐C radiation. (f) H_2_O_2_ content in plant shoot tips 3 days after UV‐C stress. (g) Level of superoxide accumulation 3 days after UV‐C stress. (h) H_2_O_2_ content in plant shoot tips 14 days after UV‐C stress. (i) Level of superoxide accumulation 14 days after UV‐C stress. Data represent values from three independent experiments (*n* = 3), and the error bar indicates standard error. For Figures A to C and F to I, two‐way ANOVAs with Tukey's multiple comparison tests were used for identifying the significant difference in values (ns = nonsignificant, *P‐value ≤0.05, **P‐value ≤0.005, ***P‐value ≤0.001, and ****P‐value ≤0.0001).

Significantly, remaining chlorophyll levels decreased in all tested halophytes shoot tips 3 days after the UV‐C irradiation (Figure 2a). Yet, while SB and AM again reached the same chlorophyll levels as the untreated control plants, VM and EL did not recover, showing a significantly lower remaining chlorophyll 14 days after UV‐C application.

Anthocyanin accumulation in plant tissue is indicative of abiotic stress. The anthocyanin level was significantly higher in halophyte shoots 3 days after UV‐C treatment (Figure 2b). Fourteen days after applying UV‐C, a decrease in the anthocyanin level was detected in SB and AM, reaching the level of the untreated control plant. In contrast, irradiated VM and EL showed a significantly higher anthocyanin level than the untreated control plants (Figure 2c).

Western blot analyses of photosynthesis and senescence‐related proteins further supported the phenological observation in the selected halophytes. The expression level of Rubisco, the most abundant protein in green tissue (Yarmolinsky et al., 2014 and references therein), significantly decreased in VM and EL shoot tissue to 40 and 46%, respectively, compared with the untreated control, 14 days after UV‐C treatment (Figure 2d). In contrast, the UV‐C‐treated SB and AM plants showed 95 and 97% of the Rubisco protein expression in the untreated control plants 14 days after UV‐C application, indicating these plants' post‐stress recovery. Similarly, the D1 protein (encoded by *psbA* transcript), a component of the reaction center of Photosystem II (Yarmolinsky et al., 2014 and reference therein), reduced to 23 and 41% in the VM and EL plants, respectively, compared with the untreated control plants, 14 days after the UV‐C treatment. In contrast, SB and AM showed 89 and 98% protein levels, respectively, compared with the untreated control plants (Figure 2d). These results demonstrated the recovery of the photosynthetic machinery in the SB and AM plants, whereas the VM and EL plants did not recover 14 days after UV‐C irradiation.

An increased level of protein autophagy‐related 8a (ATG8a) indicates enhanced senescence. Significantly enhanced senescence symptoms in shoot tissue were observed in the 239 and 344% enhanced expression of ATG8a, compared with the untreated control plants, in VM and EL, respectively, 14 days after the UV‐C treatment (Figure 2e). In contrast, the SB and AM plants showed a decrease and a slight increase in ATG8a protein expression (71 and 106%), respectively, indicating the absence of significant senescence symptoms. The pheophorbide *a* oxygenase protein (PaO, also known as accelerated cell death 1, ACD1), another senescence marker [indicating chlorophyll breakdown (Tanaka, Hirashima, Satoh, & Tanaka, 2003)], was significantly higher in the VM and EL plants (208 and 231%, respectively) than in the control plants. In contrast, the PaO protein exhibited a much lower increase in the SB and AM plants compared with the untreated control plants [123 and 137 (Figure 2e)]. These results indicate that the degradation of protein and chlorophyll was significantly higher in the VM and EL plants, leading to substantial senescence symptoms noticed via the lower pigment levels.

### ROS accumulation most likely did not cause the higher level of damage in the VM and EL plants

To examine whether the generated reactive oxygen species (ROS) were the cause of the more significant damage in the halophyte shoots exposed to UV‐C irradiation, the H_2_O_2_ and O_2_^−^ levels were detected. Three days after UV‐C irradiation, H_2_O_2_ significantly decreased in the VM plants and increased in the AM plants. In contrast, no significant change was noticed in the SB and EL plants compared with the untreated control plants (Figure 2f). Similarly, no significant modification in O_2_^−^ level was seen in the tested halophyte plants 3 days after UV‐C treatment (Figure 2g). Notably, 14 days after the stress application, O_2_^−^ and H_2_O_2_ significantly decreased in VM, whereas EL showed a significant reduction in H_2_O_2_ and an increase in O_2_^−^ radicals. In contrast, the SB and AM plants, which showed significantly fewer damage symptoms than EL and VM, exhibited a substantial increase in H_2_O_2_ and O_2_^−^ in treated plants compared with the untreated control plants (Figure 2h,i). These results indicate that ROS was likely not the cause of these plants' different UV‐C‐induced damage symptoms.

### Reduced AO activity could have been responsible for the higher accumulation of reactive carbonyl species in the *S. fruticosa,* VM and EL plants

**Figure 3:**
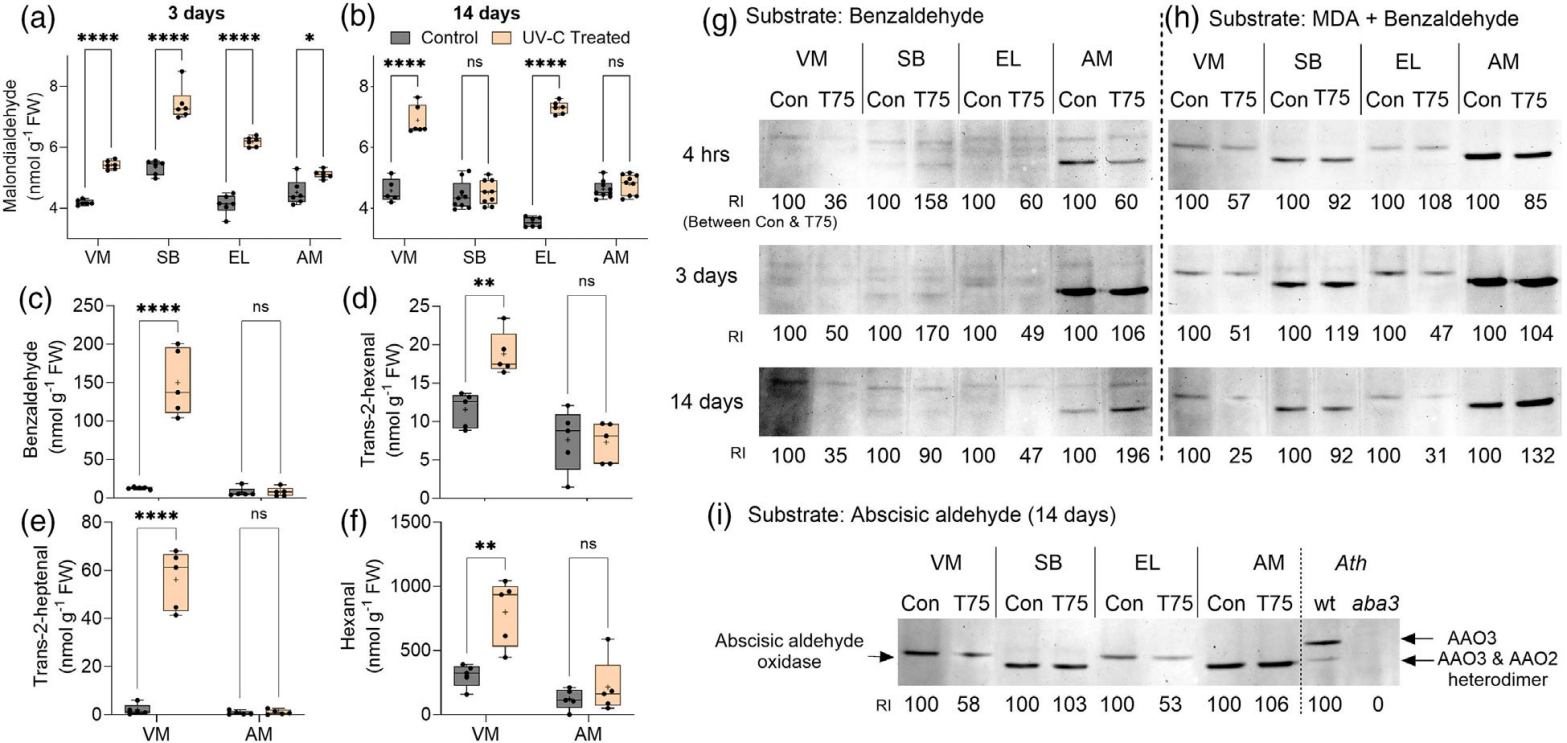
Effect of 0.075 J cm^−2^ UV‐C irradiation on aldehyde accumulation and AO activity in *Sarcocornia fruticosa* (ecotypes VM and EL), *Salicornia brachiata* (SB), and *Arthrocnemum macrostachyum* (AM). (a) Accumulation of malondialdehyde (MDA) 3 days after UV‐C stress. (b) Accumulation of MDA 14 days after UV‐C stress. Data represent at least two biological replicates from three independent experiments (*n* ≥ 6). Levels of (c) benzaldehyde, (d) trans‐2‐hexenal, (e) trans‐2‐heptenal, and (f) hexanal were identified in VM and AM plants in the control and 14 days after UV‐C treatment and are presented as box plots. Data represent five biological replicates randomly selected from three independent experiments (*n* = 5). Two‐way ANOVAs with Tukey's multiple comparison tests were used. Significant differences in values are: ns = nonsignificant, *P‐value ≤0.05, **P‐value ≤0.005, and ****P‐value ≤0.0001. (g) Aldehyde oxidase activity using 1 mM of benzaldehyde as a substrate. (h) Aldehyde oxidase activity using 3 mM of MDA + 1 mM of benzaldehyde as the substrates. (i) Aldehyde oxidase activity with 0.2 mM of abscisic aldehyde as the substrate. Relative intensity was calculated and compared with the respective control using ImageJ software (https://imagej.nih.gov/ij/). “Con” represents protein samples from control plants, and “T75” represents samples from plants 3 days after 0.075 J cm^−2^ UV‐C treatment.

The reactive carbonyl species (RCS) level was examined to determine whether RCS enhancement was the reason for the observed tissue damage. Examining the halophyte shoots 3 days after UV‐C stress revealed that the MDA level had significantly increased in all selected halophytes (Figure 3a). However, 14 days after stress application, the SB and AM shoots exhibited no significant difference in MDA content compared with the shoots of the untreated control plants (Figure 3b). In contrast, VM and EL showed significantly higher, continuously increasing MDA accumulation than the control plants 14 days after stress application. Further analyses of RCS content in the VM and AM shoot extracts revealed that 14 days after UV‐C irradiation, RCS, such as nonanal, decanal, and trans‐2‐pentenal, had not significantly changed in the UV‐C‐treated VM and AM plants, compared with the control plants (Figure S1a–c). Yet, the VM plants accumulated significantly higher levels of RCS, such as benzaldehyde, trans‐2‐hexenal, trans‐2‐heptenal, and hexanal, compared with the untreated control shoot tissue. In contrast, no enhancement was noticed in AM tissue extracts (Figure 3c–f). Previously, α,β‐unsaturated RCS was shown to be more toxic to plant cells than saturated RCS (Mano et al., 2009). Accordingly, the several‐fold increase in α,β‐unsaturated RCS, including trans‐2‐heptenal and trans‐2‐hexenal, as well as enhanced benzaldehyde and hexanal, could be the reason behind the higher toxicity, leading to tissue damage in the VM plants.

To elucidate the factor/s influencing RCS concentration, an in‐gel activity assay of AO, an enzyme recently shown to detoxify toxic aldehydes in *Arabidopsis* leaves exposed to UV‐C irradiation (Nurbekova et al., 2021, 2024), was performed. The in‐gel activity of AOs was examined in extracted halophyte shoot samples, using benzaldehyde and combined MDA with benzaldehyde as the assay substrate.

Using benzaldehyde as the substrate for AO activity revealed a decreased AO activity in the UV‐C‐treated VM and EL plants 4 h, 3 days, and 14 days after the treatment (Figure 3g). In contrast, SB showed an almost similar or increased AO activity in the UV‐C‐treated samples at all examined time points after the UV‐C treatment. As for the AM samples, except for the initial decrease in AO activity in samples taken 4 h after UV‐C irradiation, an increase in AO activity was noticed in plant shoots 3 and 14 days after UV‐C application (Figure 3g). Employing the mix of MDA and benzaldehyde as a substrate showed a clear difference in AO activity in the selected halophytes (Figure 3h). The VM and EL plants showed a clear trend of decreasing AO activity from 4 h to 14 days after the UV‐C treatment. In contrast, SB and AM showed an initial slight decrease 4 h after the UV‐C treatment and increased AO activity after 3 days. Fourteen days after UV‐C irradiation, a slight decline in the high level of AO activity was detected in SB, as well as a further increase in the AO activity of AM, compared with AO activity in the untreated control SB and AM plants, being several folds higher than AO activity in UV‐C treated VM and EL.

*Arabidopsis* aldehyde oxidase 3 (AAO3), in *A. thaliana*, is distinct from the other AOs by its specific ability to oxidize abscisic aldehyde (Seo et al., 2000; Seo et al., 2000), and it was recently shown to detoxify toxic aldehydes generated by abiotic stresses such as UV‐C irradiation (Nurbekova et al., 2021, 2024). Using abscisic aldehyde as the specific substrate was employed to verify AO activity's reliability in halophyte plants. Two activity bands of AAO3 and the AAO3 + AAO2 heterodimer in *Arabidopsis* wild‐type (wt) plants and the absence of any AAO activity in the *aba3* mutants, impaired in the molybdenum cofactor essential for AO activity, served as controls and showed similarity with previously established results (Figure 3i and Figure S1; Nurbekova et al., 2024). AO activity with abscisic aldehyde showed similar AO activity as demonstrated using the mix of MDA and benzaldehyde as the substrate; namely, there was a significant decrease in AO activity in the VM and EL plants, whereas there was a slight increase in the SB and AM plants 14 days after the UV‐C stress (Figure 3h,i).

These results support the notion that after an initial decrease in AO activity, all selected halophyte species, including SB and AM showed increased accumulation of the toxic aldehyde MDA in plant tissue after 3 days. Increased AO activity in SB and AM 3 days after the UV‐C treatment led to detoxification of aldehydes and tissue recovery. The decrease of AO activity in the VM and EL plants led to a continuous increase in the accumulation of toxic aldehydes, resulting in enhanced tissue damage.

### MDA application resulted in a similar toxicity response of the tested halophyte plants as that of UV‐C irradiation

**Figure 4:**
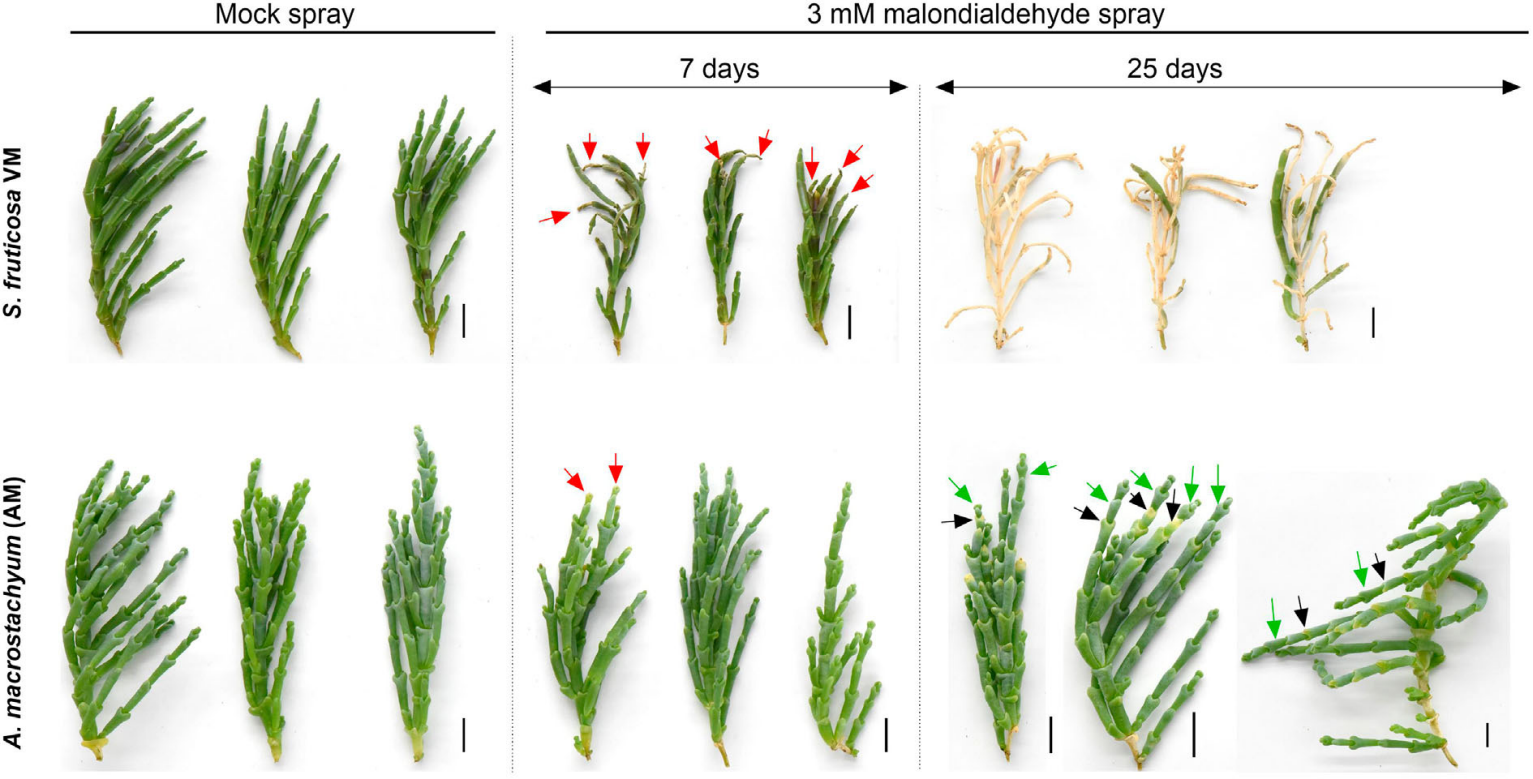
Effect of exogenously applied 3 mM of malondialdehyde (MDA) on the appearance of *Sarcocornia fruticosa* ecotype VM and *Arthrocnemum macrostachyum* (AM). Plants sprayed with a solution lacking MDA were used as a control. The red arrow shows damage on the plants 7 days after MDA spraying. Twenty‐five days after MDA spraying, the VM plants showed significant damage and mortality, whereas the AM plants showed recovery. Black arrows on the AM plants show scars of damage left behind after MDA spraying, and the green arrow shows new growth on the AM plants. Three representative plants from each treatment are presented. The scale bar represents 1 cm.

**Figure 5:**
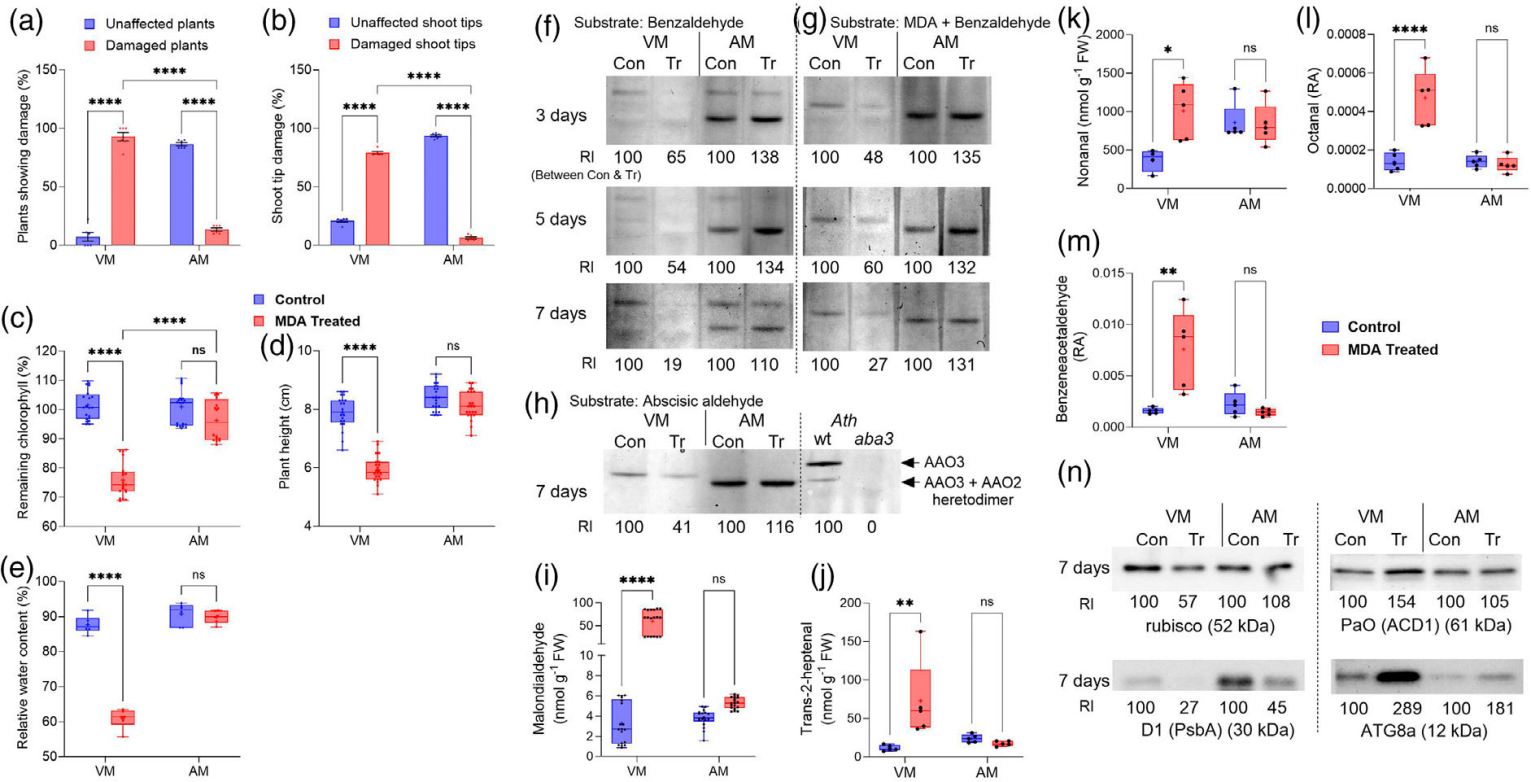
Effect of exogenously applied 3 mM of malondialdehyde (MDA) on biochemical components in *Sarcocornia fruticosa* VM and *Arthrocnemum macrostachyum* (AM). (a) Percentage of the number of plants showing surface damage in each pot. Pots containing 12 plants were used for control and MDA spraying. Six pots of each treatment were considered (*n* = 6). Seven days after the spraying, plants were examined for surface damage, including bleaching and drying of shoot tips. (b) Percentage of shoot tips damaged 7 days after MDA spraying in each pot. Data of all shoot tips, including primary and branching. Six pots for each treatment were included (*n* = 6). (c) Relative chlorophyll content 7 days after MDA spraying compared with the control plants. Data calculated using six biological replications of three independent experiments is presented (*n* ≥ 18). (d) Plant height after MDA spraying. Seven plants from three independent experiments were considered (*n* ≥ 21). (e) The relative water content of plants after MDA spraying. Data are presented using two biological replications from three independent experiments (*n* = 6). Aldehyde oxidase (AO) activity using samples collected 3, 5, and 7 days after MDA spraying. (f) AO activity using 1 mM of benzaldehyde as the substrate, and (g) AO activity using 3 mM of MDA + 1 mM benzaldehyde as the substrate, as well as (h) AO activity using 0.2 mM of abscisic aldehyde as the substrate. Chromatic bands were documented with a gel imaging system (ChemiDoc Touch imaging system, Bio‐Rad Laboratories, USA), and relative intensities were calculated, keeping the value of the respective control as 100 using ImageJ software (https://imagej.nih.gov/ij/). “Con” represents protein samples from control plants, and “Tr” represents samples treated with MDA. (i) Accumulation of MDA in response to MDA spraying. Data are represented by six biological replicates from three independent experiments (*n* = 18). The concentrations of (j) trans‐2‐heptenal and (k) nonanal and the relative abundances (RA) of (l) octanal and (m) benzeneacetaldehyde, identified in the VM and AM plants in the control and 7 days after MDA treatment, are shown as a box plot. Data represent five biological replicates randomly selected from three independent experiments (*n* = 5). (n) Western blot of photosynthesis‐related proteins Rubisco (52 kDa) and D1 (PsbA) (30 kDa) (left column), and senescence‐related proteins PaO (ACD1) (61 kDa) and ATG8a (12 kDa) (right column). ChemiDoc Touch imaging system (Bio‐Rad) recorded the blots' chemiluminescence. Image analyses and relative intensity were done as described above. Two‐way ANOVAs with Tukey's multiple comparison tests were used for identifying significant differences in values (ns = nonsignificant, *P‐value ≤0.05, **P‐value ≤0.005, and ****P‐value ≤0.0001).

The higher accumulation of an array of reactive aldehydes in VM and EL, in response to UV‐C irradiation, prompted us to examine the aldehydes' detrimental effect. The toxicity of MDA was examined by externally spraying 3 mM of MDA on the VM and AM shoots. Seven days after the spraying, it was revealed that the MDA spraying significantly damaged the VM plants. Damage observed on the VM shoot tips included (1) yellowing, indicating low damage, (2) drying of 2–3 mm of their area, indicating moderate damage, and (3) wilting and drying of more than 10 mm of their area, indicating severe damage (Figure 4). In contrast, the AM plants showed a strong resilience in response to the MDA spraying, with only a few plants showing tip yellowing. Of the total VM plants, 93% showed damage from the MDA spraying, which is significantly higher than the AM plants, of which only 14% of plants showed negative effects (Figures 4 and 5a). Similarly, 79% of the total shoot tips (including main shoot tips and branching tips) of the VM plants were affected after MDA spraying, whereas only 6% of the AM shoot tips were affected (Figure 5b). Remaining chlorophyll was significantly reduced in the VM plants 7 days after MDA spraying, whereas there was no significant change in the remaining chlorophyll in the AM plants after MDA application (Figure 5c).

Additionally, MDA application hampered plant growth and reduced plant height in VM. In contrast, the AM plants were hardly damaged by the MDA spraying, and plant height did not exhibit a significant difference between the control and the treated plants (Figure 5d). Similarly, the relative water content was significantly reduced in the VM plants 7 days after MDA spraying, whereas the treated AM plants maintained similar relative water content as the control plants (Figure 5e). In the long term (25 days after the treatment), the VM plants dried out, exhibiting mortality, whereas the AM plants recovered and showed growth of new branches (Figure 4). Interestingly, O_2_^−^ and H_2_O_2_ accumulation levels were significantly reduced in the VM plants treated with 3 mM of MDA compared with the control plants, whereas the AM plants showed a significant ROS increase in the treated plants (Figure S2a,b). These results further reduce the likelihood that ROS was the cause of the damage in the MDA‐treated VM plants.

AO in‐gel activity was detected using shoots sampled 3, 5, and 7 days after MDA spraying, using benzaldehyde with or without MDA as the AO activity substrate (Figure 5f,g). The VM plants showed significantly lower AO activity than the untreated control shoots at all time points tested, with the lowest activity found in shoots sampled 7 days after the spraying. In contrast, compared with the activity in the untreated control shoots, AO activity in the AM shoots sprayed with MDA increased in the samples taken 3, 5, and 7 days after the spraying, using benzaldehyde or a mix of benzaldehyde and MDA as the substrate (Figure 5f,g). Similarly, employing abscisic aldehyde as the substrate resulted in decreased AO activity in the VM plants, whereas increased activity 7 days after MDA spraying was noticed in the AM shoots (Figure 5h). Accordingly, the estimation of changes in the RCS level in MDA‐treated plants revealed 18‐fold higher MDA levels in the VM plants than in the untreated control plants. In contrast, the AM plants showed a slightly insignificant MDA content increase (Figure 5i). Similarly, aldehydes, such as trans‐2‐heptenal, nonanal, octanal, and benzeneacetaldehyde, were significantly higher in the MDA‐treated VM plants than in the control plants. In contrast, no significant change was noticed in the AM plants (Figure 5j–m), whereas some aldehydes did not significantly change in either the VM or the AM plants (Figure S2c–h).

Previously, it was shown that short‐chain RCS, including ketones such as acetone, increase under salinity stress as a secondary effect (Sultana et al., 2024). The MDA treatment resulted in a significantly increased relative abundance of the α, β‐unsaturated ketone 6‐methyl, 5‐hepten‐2‐one in the VM plants, whereas the AM plants did not show any changes (Figure S3a). Similarly, α, β‐unsaturated ketones, such as 6‐methyl, 5‐hepten‐2‐one, and 3‐octen‐2‐one, significantly increased in the VM plants 14 days after UV‐C stress, whereas no significant changes were noticed in the AM plants (Figure S3b,c). Other ketones, including β‐ionone, 2‐butanone, and 2‐pentanone, remained unchanged in the UV‐C and MDA‐treated VM and AM plants (Figure S3d–i).

Damage caused by accumulated RCS in the VM plants 7 days after MDA spraying was further evaluated by Western blot analyses of photosynthesis and senescence‐related proteins (Figure 5n). The expression of both Rubisco and D1(PsbA) proteins was lower in the MDA‐sprayed VM plants than in the control plants. In contrast, only the D1 protein showed lower expression in the MDA‐treated AM plants than in the control plants, yet higher expression than in the MDA‐treated VM. Additionally, compared with the control plants, the senescence‐related proteins ATG8a and PaO significantly increased in the MDA‐treated VM and AM plants, being lower in the AM than in the VM plants and thus supplying further confirmation that the VM plants are more susceptible than the AM plants, exhibiting more significant damage in response to the MDA spraying.

### Benzaldehyde application resulted in a similar toxicity response of the tested halophyte plants as that of MDA spraying or UV‐C irradiation

**Figure 6:**
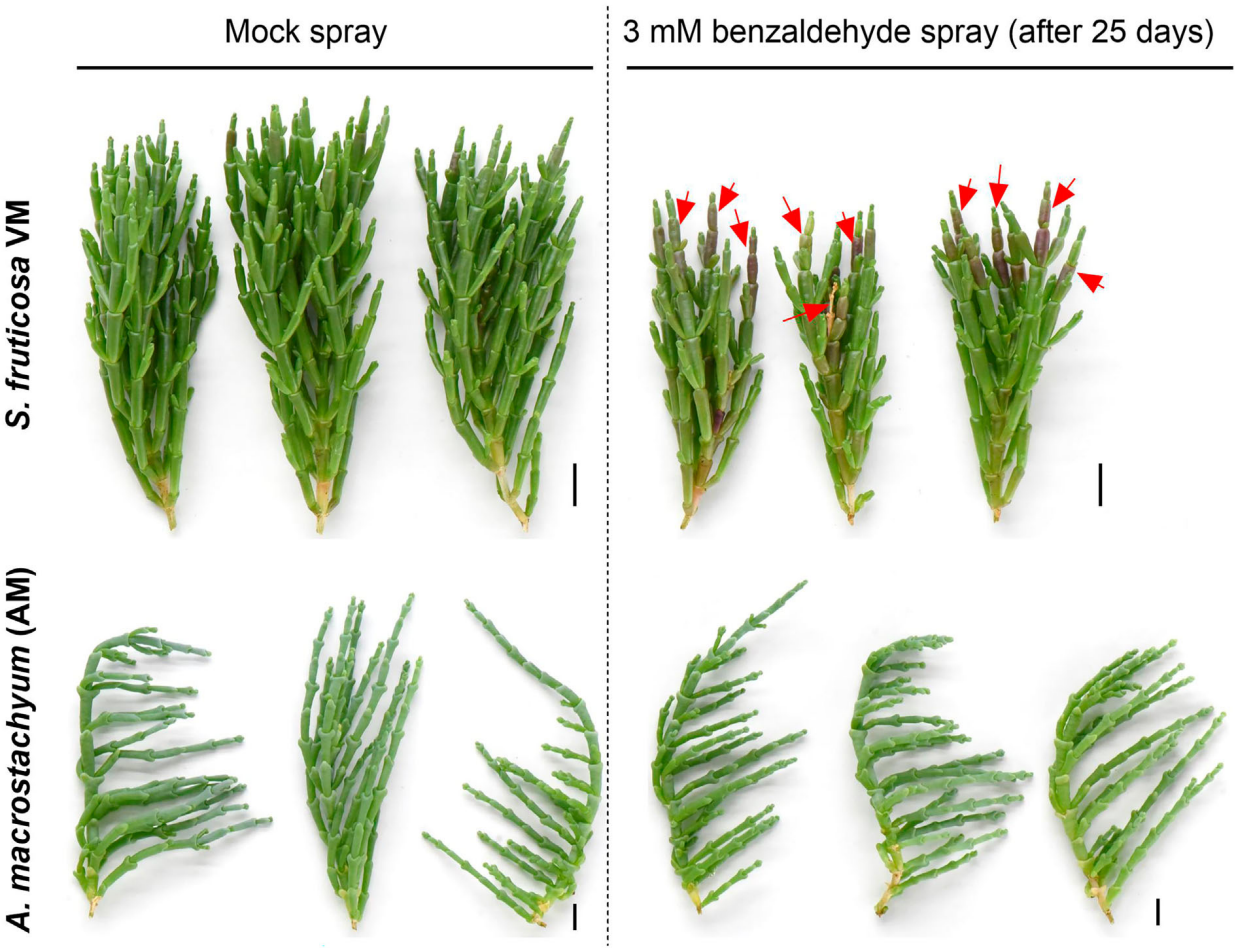
Effect of exogenously applied benzaldehyde on the appearance of *Sarcocornia fruticosa* ecotype VM and *Arthrocnemum macrostachyum* (AM). Plants sprayed with a solution that did not contain benzaldehyde were used as a control. Plants were photographed 25 days after the control spraying and the 3 mM‐benzaldehyde spraying. Red arrows indicate damage to shoot tips and visible red color of anthocyanin accumulation in VM plants. The scale bars represent 1 cm.

**Figure 7:**
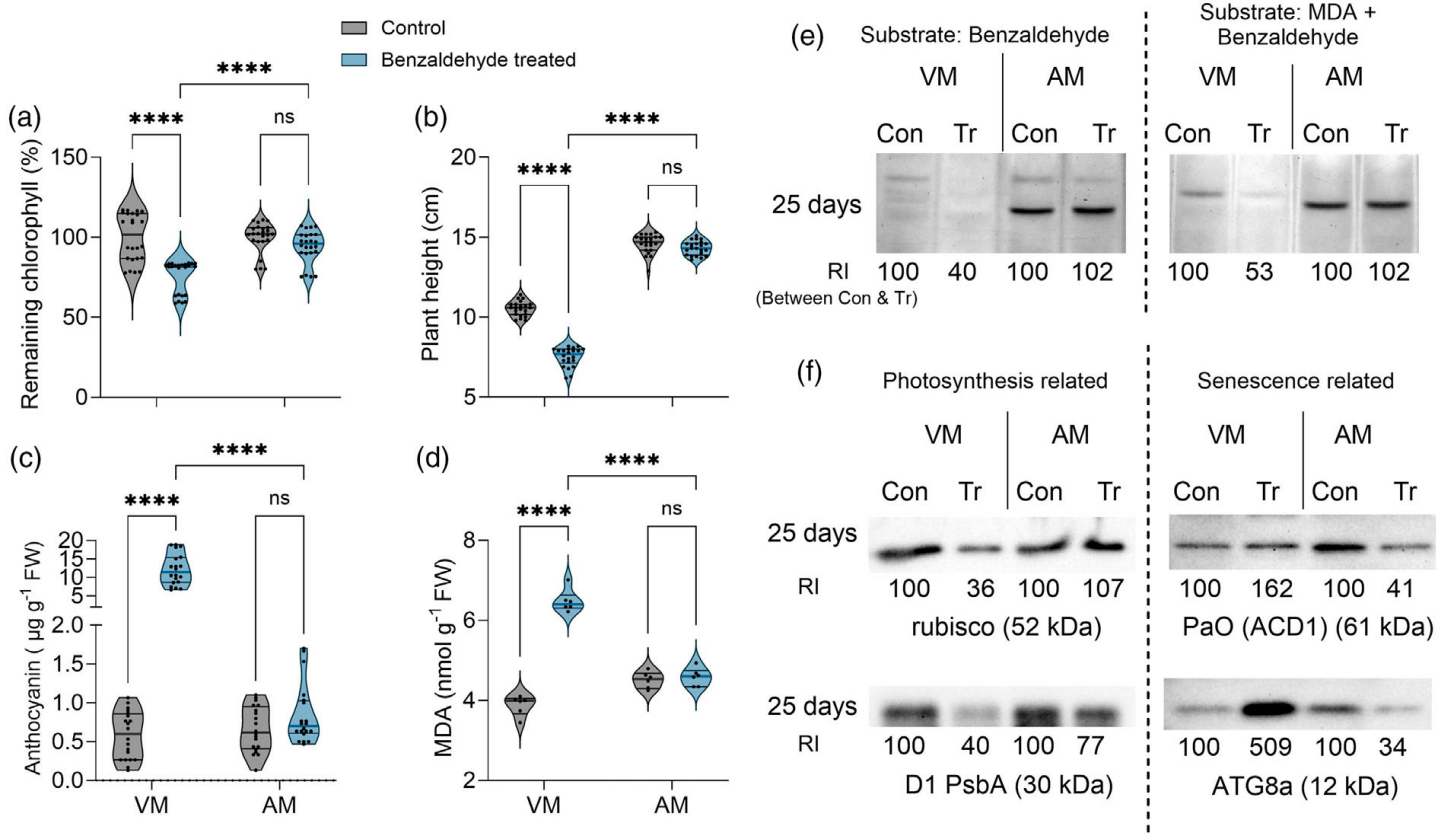
Effect of exogenously applied 3 mM of benzaldehyde on biochemical components in *Sarcocornia fruticosa* VM and *Arthrocnemum macrostachyum* (AM). (a) Remaining chlorophyll 25 days after benzaldehyde spraying. Data represent eight biological replicates from three independent experiments (*n* = 24). (b) Plant height 25 days after spraying. Heights of at least seven plants from three independent experiments were considered (*n* ≥ 21). (c) Truncated violin plot showing anthocyanin accumulation in shoot tips 25 days after spraying. Data represent at least six biological replicates from three independent experiments (*n* ≥ 18). (d) MDA accumulation in shoot tips 25 days after spraying. Data represent two biological replicates from three independent experiments (*n* = 6). Violin plot shows 25th and 75th percentile, with a thin line and a thick line indicating the median in (a–d). Two‐way ANOVAs with Tukey's multiple comparison tests were used for identifying significant differences in values (ns = nonsignificant and ****P‐value ≤0.0001). (e) Aldehyde oxidase (AO) activity using 1 mM of benzaldehyde as the substrate in the left column and activity with 3 mM of MDA + 1 mM benzaldehyde as the substrate in the right column. The relative intensities of chromatic activity bands were calculated by keeping the respective control value as 100 using ImageJ software (https://imagej.nih.gov/ij/). When using benzaldehyde as a substrate, two or more bands appeared in different species. The sum of the intensity of all bands was used for relative intensity calculation. “Con” and “Tr” represent samples from plants treated with the control and the benzaldehyde spray, respectively. (f) Western blot of photosynthesis‐related proteins Rubisco (52 kDa) and D1 (PsbA) (30 kDa) are presented in the left column, and the senescence‐related proteins PaO (ACD1) (61 kDa) and ATG8a (12 kDa) in the right column. The bands' relative intensities were calculated between the control and the treated samples, using ImageJ software (https://imagej.nih.gov/ij/). “Con” and “Tr” represent samples from plants treated with the control and the benzaldehyde spray, respectively.

To verify that the plant responded to UV‐C irradiation similarly to toxic aldehydes, the effect of 3 mM of benzaldehyde spray was also tested and revealed that the VM plants showed significantly hampered growth and anthocyanin accumulation on shoot tips 25 days after the application (see red arrows in Figure 6). Subsequently, significant reductions in relative chlorophyll and plant height were noted in the VM plants, whereas the AM plants showed no difference compared with the control plants (Figure 7a,b). As observed in the treated shoots (Figure 6), the anthocyanin level was significantly higher in the benzaldehyde‐sprayed VM plants than in the control plants, whereas no significant difference was noticed in the AM‐treated plants compared with the control plants (Figure 7c). Similarly, the MDA level was significantly higher in the sprayed VM plants than in the control plants, whereas no significant difference was noticed in the AM plants sprayed with benzaldehyde compared with the control plants (Figure 7d).

Examination of AO activity 25 days after benzaldehyde spraying showed significantly reduced AO activity in the benzaldehyde‐sprayed VM plants compared with the control plants with both substrates: benzaldehyde and MDA + benzaldehyde (Figure 7e). In contrast, a slight increase in AO activity was observed in the benzaldehyde‐treated AM plants compared with the control plants. Most likely, due to the lower AO activity, MDA accumulations were significantly higher in the benzaldehyde‐sprayed VM plants but not in the AM plants (Figure 7d). Western blot analyses of Rubisco and D1 proteins showed significant reductions in the VM plants compared with the control plants 25 days after benzaldehyde spraying, while the AM plants did not show a decrease in the Rubisco protein but did show a reduction in the D1 protein, which was less than in the VM plants (Figure 7f). Compared with the control plants, the accumulation of the senescence‐related proteins ATG8a and PaO significantly increased in the benzaldehyde‐treated VM plants. In contrast, these proteins were less expressed in the treated AM plants than in the control plants (Figure 7f). These results confirmed that the spraying of benzaldehyde, which was shown to be enhanced as the result of UV‐C irradiation (Figure 3f), indeed had a toxic effect on the VM plants, resulting in the accumulation of additional cytotoxic RCS. In contrast, the AM plants could detoxify the exogenously applied aldehyde by enhancing the AO activity. In summary, the exogenous application of MDA and benzaldehyde, shown to accumulate in EL and VM in response to UV‐C irradiation, caused a similar toxicity as UV‐C irradiation in the VM but not in the AM plants, due to enhanced AO activity that was absent in the VM plants.

## DISCUSSION

ROS play a significant role in plant abiotic stress signaling (Peláez‐Vico, Fichman, Zandalinas, Foyer, & Mittler, 2024) and are primary damage‐causing agents during plant abiotic stress (Choudhury, Rivero, Blumwald, & Mittler, 2017). Yet, it is becoming more apparent that ROS are not always directly involved in damage to plant cells during abiotic stress (Mittler, 2017; Xu, Liu, Mittler, & Shabala, 2024). Accordingly, in the current study, the stress‐susceptible halophytes, the VM and EL plants, mainly showed a decrease in ROS content but an increase in RCS content while the stress‐resistant halophytes, the SB and AM plants, exhibited an increase in ROS content but no change in RCS content (Figures 2f–i, 3a–f and 5i–m; Figures S2 and S3). As evidence, it was recently shown that RCS accumulation was responsible for *Arabidopsis* root growth retardation rather than ROS (Sultana et al., 2024). Further, studies have emphasized the role of RCS in regulating PCD in plants. Acrolein and 4‐hydroxy‐(E)‐2‐nonenal were shown to activate caspase‐like proteases, triggering oxidative signal‐induced PCD (Biswas & Mano, 2016). Similarly, (E)‐2‐hexenal enhanced caspase‐3‐like activity during aluminum‐induced PCD in wheat roots, suggesting lipid peroxidation‐derived aldehydes mediate cell death (Liang et al., 2023). Furthermore, elevated RCS levels following oxidative stress or γ‐radiation exposure were linked to PCD and stress adaptation mechanisms (Ksas et al., 2024; Qian et al., 2025), highlighting RCS as crucial signals connecting oxidative stress to cell death pathways.

*Arabidopsis* aldehyde oxidase 3 (AAO3) has demonstrated the detoxification of more than 20 different carbonyl aldehydes, including benzaldehyde and MDA (MDA) (Nurbekova et al., 2021). Similarly, *Arabidopsis* aldehyde oxidase 4 (AAO4) has demonstrated the detoxification of various aldehydes, primarily benzaldehyde in siliques (Ibdah, Chen, Wilkerson, & Pichersky, 2009; Srivastava et al., 2017). Further, *Arabidopsis aao3* and *aao4* knockout mutants confirmed the role played by these enzymes in the detoxification of RCS in *Arabidopsis* leaves and siliques under UV‐C irradiation, Rose Bengal, and exogenously applied aldehyde stresses (Nurbekova et al., 2021, 2024; Srivastava et al., 2017). Unlike in glycophytic plants, RCS toxicity and aldehyde detoxification are rarely studied in halophytes. In the current study, in‐gel activity, with halophyte crude protein extract, using abscisic aldehyde as the substrate, which was previously shown to be a specific substrate for *Arabidopsis* AO3 (Seo, Koiwai, et al., 2000; Seo, Peeters, et al., 2000) that detoxifies toxic aldehydes (Nurbekova et al., 2021, 2024), clearly showed AO activity enhancement in the more resistant AM and SB halophytes. In contrast, the susceptible halophytes, the EL and VM plants, exhibited a decrease in AO activity in response to UV‐C irradiation (Figure 3i).

Interestingly, exogenous application of benzaldehyde, nonanal, hexanal, or cinnamaldehyde increased internal RCS levels, including propionaldehyde, HNE, benzaldehyde, hexanal, acrolein, and cinnamaldehyde, leading to chlorosis and senescence in wild‐type and *aao3* mutant *Arabidopsis* plants (Nurbekova et al., 2021). Similarly, exogenously applied benzaldehyde, citral, hexanal, and naphthaldehyde on the *aao4* knockout mutant increased the MDA level, decreased the chlorophyll level, and resulted in enhanced senescence symptoms, whereas the wt and AAO4 OE lines hardly showed any symptoms (Srivastava et al., 2017). These results highlight aldehyde oxidases' vital role in detoxifying increased internal RCS. Notably, similar to UV‐C irradiation, the current study confirmed RCS toxicity in the selected halophyte plants by exogenous application of MDA or benzaldehyde to the halophytes (Figures 4 and 6). The VM plants showed drastic effects of the MDA spraying, including reduced shoot length, chlorophyll, and relative water contents (Figure 5c–e) and significantly higher shoot damage, including mortality, 25 days after the application (Figure 4). In contrast, the AM plants tolerated MDA toxicity and recovered after initial symptoms (Figure 4). Further, the VM plants showed significantly enhanced accumulation of endogenous RCS, MDA, nonanal, benzeneacetaldehyde, octanal, and the highly toxic α, β‐unsaturated RCS, the trans‐2‐heptenal and 6‐methyl, 5‐hepten‐2‐one, 7 days after the treatment (Figure 5i–m and Figure S3).

Notably, similar results of a decrease in remaining chlorophyll and shoot height, as well as enhanced toxic MDA levels in shoot extracts, and significant damage in chloroplast proteins, in response to benzaldehyde spray, were noticed in the VM plants 25 days after the benzaldehyde spraying. In contrast, the AM plants were unaffected (Figure 6), further supporting AM's enhanced resistance to the applied environmental stresses. Significantly, similar to the response to UV‐C irradiation, AO activity increased in the AM plants in response to the application of either MDA or benzaldehyde, whereas in the VM plants sprayed with either of the two aldehydes, AO activity decreased (Figures 5f–h and 7e), indicating that the AO activity level plays a role in detoxifying toxic aldehydes in the shoots of the examined halophytes.

## METHODS

### Preparation of plant material

Seeds of the edible halophyte *Sarcocornia fruticosa* [ecotypes VM and EL (Ventura et al., 2011; Ventura & Sagi, 2013)], *Salicornia brachiata* [SB (Patel et al., 2024)], and *Arthrocnemum macrostachyum* [AM (Sisay et al., 2022)] were first placed in a tap water‐wetted sterile potting mix for 1 week. Germinated seeds grew in a greenhouse for 1 month in the same pot (12 cm length × 8 cm width × 6 cm depth). Seedlings were initially irrigated with tap water for 10 days and later gradually irrigated using water containing increasing NaCl levels, up to 100 mM, including 1 g/L NPK plus microelements (20:20:20; Haifa Chemicals, Haifa, Israel), equivalent to 15 mM nitrogen, for 20 days. Twelve seedlings per halophyte type were transferred to a new pot containing sterile soil (two rows of six plants). Seedlings were allowed to grow for two additional months and irrigated with water containing 100 mM of NaCl and 1 g/L of NPK fertilizer every 2–3 days. Plants were grown in controlled greenhouse conditions at Midreshet Ben‐Gurion, Israel (30.8523° N, 34.7834° E). Growth conditions in the greenhouse included midday PAR that reached 650–700 μmol/m^2^/s and controlled temperatures (from 25 to 35°C).

*Arabidopsis thaliana* var. Columbia wild‐type (WT) and *aba3‐1* (AT1G16540) mutants (Bekturova et al., 2021; Sagi, Scazzocchio, & Fluhr, 2002) were grown in a controlled growth room (14 h 150 μmol m^−2^ sec^−1^ light/10 h dark, 22°C, with 75–85% relative humidity). Leaf samples were harvested after 25 days and were used as an additional control in the in‐gel activity assays.

### UV‐C and aldehyde plant treatments

Nighty‐day‐old plants were divided into two groups, one for the UV‐C treatment and the second for the control. Plants were treated with 0.075 J cm^−2^ UV‐C irradiation in a microprocessor‐controlled UV radiation system and then transferred back to the greenhouse after UV‐C treatment. The top 2 cm of the main shoot tips were sampled from the respective control and the UV‐C treated plants at different time points after UV‐C treatment. Samples were taken after 4 h, 3 days, and 14 days, and were immediately transferred into liquid nitrogen and stored at −80°C. For phenotyping, a 5‐cm‐long main shoot tip was harvested, and photos were taken with a white background using a Nikon D750 DSLR equipped with AF‐S Micro Nikkor 105 mm 1:2.8 G lens.

Next, 90‐day‐old VM and AM plants that varied in their resistance to UV‐C irradiation were thoroughly sprayed with 3 mM of MDA and 3 mM of benzaldehyde in distilled water containing 0.01% Tween 20. Distilled water containing 0.01% Tween 20 was sprayed onto the control plants. The aldehydes were sprayed three times (every other day) onto the same plants. Seven days after the first spraying, the plants were thoroughly washed, first with 0.01% Tween 20 and then with distilled water, and 2‐cm shoot tip sections were harvested and stored at −80°C. Phenotyping was performed similarly to the process mentioned above for the UV‐C treatment.

### Determination of ROS content

For H_2_O_2_ detection, shoot tip samples were extracted in a 1:8 (w/v) ratio of chilled 100 mM potassium phosphate buffer (pH 7.5). A 40‐μl plant extract was added to the reaction mixture containing 100 μl 100 mM potassium phosphate buffer (pH 7.5), 20 μl 8.5 mM 4‐amino antipyrine, 20 μl 34 mM sodium 3,5‐dichloro‐2‐hydroxybenzenesulfonate, and 20 μl 45‐unit mL^−1^ horseradish peroxidase (HRP). The 100 mM potassium phosphate buffer (pH 7.5) replaced the plant extract for the blank reaction. HRP was added to the reaction mixture to initiate the reaction, and 10 min later, the absorbance was taken at 500 nm. A standard curve (ranging from 0 to 200 μm H_2_O_2_) was prepared with 1 mM of H_2_O_2_ solution and was used to calculate H_2_O_2_.

In the shoot tip samples, superoxide radicals were detected by the reduction of 2,3‐bis [2‐methoxy‐4‐nitro‐5‐sulfophenyl 2H‐tetrazolium‐5‐carboxanilicle (XTT)]. Shoot tip samples were crushed in extraction buffer (100 mM potassium phosphate, pH 6.0, 0.5 mM XTT sodium salt) in a 1:10 volume ratio and then incubated at 40°C in dark conditions for 2 h. After the incubation, the tubes were centrifuged, and the supernatant was used for absorbance measurement at 470 nm.

### Chlorophyll and anthocyanin estimation

For chlorophyll extraction, 100 mg of shoot tip tissue was crushed in 80% ethanol in a 1.5‐mL centrifuge tube; for complete chlorophyll extraction, tubes were stored at 4°C for 48 h with intermittent shaking. After 48 h, the tubes were centrifuged, and the supernatant was used for the absorbance measurements at 649 and 665 nm. Total chlorophyll was estimated according to Nurbekova et al. (2021).

For anthocyanin extraction, shoot tips were extracted in a 1:8 (w/v) ratio of cold methanol containing 1% HCl and kept on ice for 30 min. Tubes were centrifuged (15 000 ***g***), and 500 μl of the supernatant was mixed with 500 μl of water and 1 mL of chloroform and vortexed thoroughly. Tubes were centrifuged at 15 000 ***g*** for 20 min, and the upper aqueous phase was used for absorbance at 530 and 657 nm for anthocyanin content estimation, following Sisay et al. (2022).

### Estimation of reactive carbonyl species

MDA content was estimated using the colorimetric method. Samples were extracted in a 1:10 (w/v) ratio of chilled extraction buffer [1% trichloroacetic acid (TCA), 0.1 M phenylmethylsulfonyl fluoride (PMSF) in a phosphate‐buffered saline solution]. Plant extracts were divided into two sets and mixed with an equal amount of 10% TCA and 0.8% thiobarbituric acid (TBA) in 10% TCA, respectively. The extraction buffer was replaced with plant extract to create a blank reaction. Tubes were heated to 99°C for 1 h, and the absorbance of the developed color was taken at 440, 532, and 600 nm. Calculating MDA content was performed according to Hodges, DeLong, Forney, and Prange (1999). The reaction with 10% TCA acts as an additional control to remove the effect of anthocyanin and sugar complex accumulation.

Other reactive carbonyl species (RCS) were detected by Headspace Solid‐Phase Microextraction (SPME), coupled with GC–MS, following Delory, Delaplace, du Jardin, and Fauconnier (2016) with certain modifications. First, 1 g of the shoot tip tissue was homogenized in a liquid nitrogen‐cooled mortar pestle and added to the 20‐mL amber glass vial containing 1 g NaCl. Then, 5 mL of an enzymatic activity stopping buffer (5 M NaCl and 0.1 M EDTA) and 10 ppm of isobutyl benzene (internal standard) were added to the tube and immediately sealed with a metallic magnetic cap containing PTFE septa. Samples were incubated at 60°C for 15 min to equilibrate the vial headspace with volatile compounds in a PAL COMBI‐xt autosampler (CTC Analytics AG, Zwingen, Switzerland). Before extraction, a DVB/CWR/PDMS SPME fiber (10 mm length, 80 μm thickness) (PALsysetm; CTC Analytics AG) was preconditioned according to the manufacturer's instructions. Volatiles were desorbed at 250°C for 10 min in a splitless injector. Separation of volatiles was achieved with an Agilent 7890A/5977B GC–MS (Agilent Technologies, Santa Clara, CA, USA) equipped with a 30 m length, 0.25 mm inner diameter, 0.25 μm film thickness + 10 m EZ‐Guard (VF‐5 ms; Agilent Technologies). Helium was used as the carrier gas at a flow rate of 1.0 mL/min. The column temperature was held at 35°C for 2 min and then programmed at 6°C/min to 250°C and held for 3 min. MS conditions were as follows: an ion source, 200°C; electron energy, 70 eV; quadrupole temperature, 150°C; GC–MS transfer line, 280°C; scan range, 40–400 mass units. An alkane standard mixture (C10‐C40) (Sigma‐Aldrich, St. Louis, MO, USA) was used as a retention index calibration standard. The exact concentrations of aldehydes in the plant samples were calculated from the standard curve of benzaldehyde, *trans*‐2‐pentenal, trans‐2‐heptenal, decanal, nonanal, trans‐2‐hexenal, trans‐2‐nonenal, and hexanal. Other aldehydes and ketones were identified and confirmed from the NIST mass spectral database and calculated as a relative abundance. The chemicals used in this procedure were HPLC grade and purchased from Sigma‐Aldrich unless specified.

### Western blotting

For Western blotting, total proteins were extracted from shoot tip tissues using an extraction buffer containing 200 mM of Tris‐HCl, pH 8.48, 1 mM of EDTA, 15 mM of reduced glutathione, 50 mM of DTT, 5 mM of l‐cysteine, 0.5 mM of sodium molybdate, 20 μm of PMSF, 1 μm of pepstatin, 10 μm of leupeptin, 0.1 μm of aprotinin, and 250 mM of sucrose. The protein content in the samples was estimated and equalized using the Bradford reagent (Bio‐Rad Laboratories, Hercules, CA, USA). Then, 15 μg of protein for ATG8a and PaO, 0.25 μg for Rubisco large subunit, and 20 μg for the D1 protein, along with a protein marker (PM2500; Bio‐Lab Ltd., Jerusalem, Israel), were separated using a 1.5 mm thick and 12.5% Denaturing, Discontinuous Polyacrylamide Gel Kit (Bio‐Rad Laboratories), following Laemmli, 1970. Proteins were transferred to a polyvinylidene fluoride (PVDF) membrane, blocked with skimmed milk, and incubated with appropriate primary and HRP‐conjugated secondary antibodies sequentially. The primary antibodies of Rubisco large subunit [a gift from Prof. Michal Shapira (Life Science Department, Ben‐Gurion University of the Negev, Israel), D1 (Anti‐PsbA; Agrisera AB, Vännäs, Sweden), ATG8a (Anti‐ATG8A‐I; Agrisera AB), and PaO (Anti‐ACD1; Agrisera AB)] were diluted to 1:20 000, 1:10 000, 1:4000, and 1:5000, respectively. The secondary antibody (anti‐rabbit IgG; Sigma‐Aldrich) was diluted to 1:12 000 in PBS. Blots were treated with the Clarity Western ECL substrate and documented in the ChemiDoc Touch imaging system (Bio‐Rad Laboratories). Image analyses and relative intensity calculations were performed with Image Lab (Bio‐Rad Laboratories) and ImageJ software (https://imagej.nih.gov/ij/).

### Aldehyde oxidase (AO) enzymatic activity in electrophoretic gels

Total proteins were extracted from shoot tip tissues using an extraction buffer, as described in the Western blotting procedure. Two hundred μg of total protein per lane was loaded and separated on a 7.5% non‐denaturing, discontinuous 1.5 mm thick polyacrylamide gel and separated under cold conditions. Thereafter, gels were incubated in a buffer containing 50 mM of Tris–HCl, pH 7.4, 0.5 mM of MTT, 0.1 mM of PMS, and a substrate (1 mM of benzaldehyde and/or 3 mM of MDA, or 0.1 mM of abscisic aldehyde). The chromatic bands that appeared due to AO activity were recorded in the gel imaging system. Image analyses and relative intensity calculations were performed with Image Lab and ImageJ software (https://imagej.nih.gov/ij/). When benzaldehyde was used as a substrate, two or more bands appeared in different species. The sum of the intensity of all bands was used for relative intensity calculation.

### Statistical analysis

Each experiment was performed at least three times. Statistical analyses were performed with IBM SPSS 26 (IBM Corporation, Armonk, New York, USA) and GraphPad Prism 10 (GraphPad Software, Boston, MA, USA). Two‐way ANOVAs with Tukey's multiple comparison tests were used to identify the significant value differences. Simultaneously, only those values passing the Kolmogorov–Smirnov (distance) test for normality were considered for analysis. Plots were prepared with GraphPad Prism 10. Images recorded from the in‐gel documentation system (ChemiDoc Touch imaging system; Bio‐Rad Laboratories) were processed through Image Lab 5.2.1 software (Bio‐Rad Laboratories). The relative intensities of the images of the in‐gel electrophoretic activity assay and the Western blotting assay were calculated using ImageJ software (https://imagej.nih.gov/ij/). MassHunter Qualitative Analysis, version B.07.00 (Agilent Technologies), and MassHunter Quantitative Analysis, version 10.0 (Agilent Technologies), were used to calculate the concentration or relative abundance of the compounds in SPME‐GC–MS.

## ACCESSION NUMBERS

*Arabidopsis* sequence data for *aba3‐1* mutant can be found in GenBank or TAIR (https://www.arabidopsis.org/) with accession number AT1G16540.

## AUTHOR CONTRIBUTIONS

JP and KK conceived the idea, participated in designing the research plans, and performed the experiments and data analysis; TAS, BC, AM, VM, and DS participated in the preparation of plant material; ZN and ZDN participated in the in‐gel activity and figure preparation; AM and NS participated in the SPME‐GC–MS analysis. MS conceived the idea, designed the research plan, and supervised the research work. JP, KK, and MS wrote and edited the manuscript. All authors approved the final manuscript.

## Supporting information


**Figure S1.** Effect of 0.075 J cm^−2^ UV‐C irradiation on aldehyde accumulation and AO activity in *Sarcocornia fruticosa* (ecotypes VM and EL), *Salicornia brachiata* (SB), and *Arthrocnemum macrostachyum* (AM). The concentration of (a) nonanal, (b) decanal, and (c) trans‐2‐pentenal in the VM and AM plants in the control and 14 days after UV‐C treatment. Data represent five biological replicates randomly selected from three independent experiments with similar results (*n* = 5). Two‐way ANOVAs with Tukey's multiple comparison tests were used to identify significant differences in values (ns = nonsignificant). (d) Confirmation of aldehyde oxidase (AO) activity in selected halophyte plants using *Arabidopsis* wild‐type and *Arabidopsis aba3* mutants employing different activity substrates. Chromatic bands were allowed to develop for 6 h and documented with a gel imaging system (ChemiDoc Touch imaging system; Bio‐Rad Laboratories, USA). *Ath* wt = *Arabidopsis thaliana* wild‐type, *Ath aba3* = *Arabidopsis thaliana aba3* mutant.

**Figure S2.** Effect of exogenously applied 3 mM of malondialdehyde (MDA) on ROS and aldehyde levels in *Sarcocornia fruticosa* VM and *Arthrocnemum macrostachyum* (AM) 7 days after the application. (a) Level of superoxide accumulation 7 days after MDA application, (b) H_2_O_2_ content in plant shoot tips 7 days after MDA application. Data represent three biological replicates from three independent experiments (*n* = 9), and the error bar indicates standard error. Concentration of (c) benzaldehyde, (d) decanal, (e) trans‐2‐nonenal, (f) trans‐2‐pentenal, (g) trans‐2‐hexenal, and (h) hexanal in the control and 7 days after MDA application. Data represent five biological replicates randomly selected from three independent experiments (*n* = 5). Two‐way ANOVAs with Tukey's multiple comparison tests were used for identifying significant differences in values (ns = nonsignificant, *P‐value ≤0.05, ***P‐value ≤0.001, and ****P‐value ≤0.0001).

**Figure S3.** Relative abundance (RA) of identified ketones in *Sarcocornia fruticosa* (VM) and *Arthrocnemum macrostachyum* (AM) exposed to UV‐C irradiation and MDA spray. (a) 6‐methyl, 5‐hepten‐2‐one in 3‐mM‐MDA‐treated plants. (b) 6‐methyl, 5‐hepten‐2‐one and (C) 3‐octen‐2‐one in UV‐C‐treated plants. (d) β‐ionone, (e) 2‐butanone, and (f) 2‐pentenone in MDA‐treated plants. (g) β‐ionone, (h) 2‐butanone, and (i) 2‐pentenone in UV‐C treated plants. Two‐way ANOVAs with Tukey's multiple comparison tests were used for identifying significant differences in values (ns = nonsignificant and ****P‐value ≤0.0001).

## Data Availability

The data that supports the findings of this study are available in the supplementary material of this article.
