# Supplementary figures and images for: UV‐C‐induced reactive carbonyl species are better detoxified in the halophytic plants Salicornia brachiata and Arthrocnemum macrostachyum than in the halophytic Sarcocornia fruticosa plants
Source: Plant J. 2025 May 27;122(4):e70239. doi: 10.1111/tpj.70239 (PMC12113106; doi:10.1111/tpj.70239)

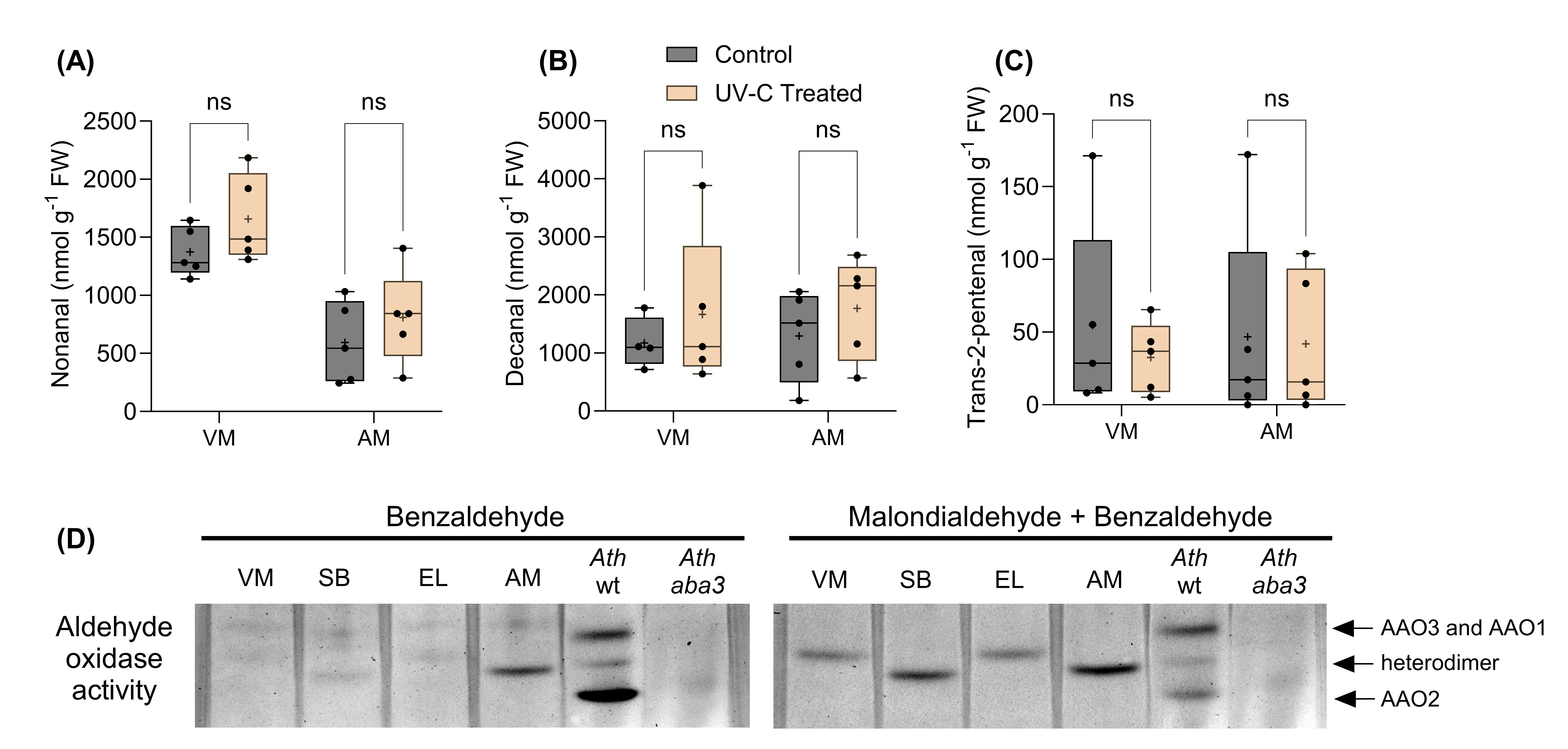

Supplement: Supplementary file 1 — Figure S1. Effect of 0.075 J cm−2 UV‐C irradiation on aldehyde accumulation and AO activity in Sarcocornia fruticosa (ecotypes VM and EL), Salicornia brachiata (SB), and Arthrocnemum macrostachyum (AM). The concentration of (a) nonanal, (b) decanal, and (c) trans‐2‐pentenal in the VM and AM plants in the control and 14 days after UV‐C treatment. Data represent five biological replicates randomly selected from three independent experiments with similar results (n = 5). Two‐way ANOVAs with Tukey's multiple comparison tests were used to identify significant differences in values (ns = nonsignificant). (d) Confirmation of aldehyde oxidase (AO) activity in selected halophyte plants using Arabidopsis wild‐type and Arabidopsis aba3 mutants employing different activity substrates. Chromatic bands were allowed to develop for 6 h and documented with a gel imaging system (ChemiDoc Touch imaging system; Bio‐Rad Laboratories, USA). Ath wt = Arabidopsis thaliana wild‐type, Ath aba3 = Arabidopsis thaliana aba3 mutant. [file TPJ-122-0-s002.jpg]

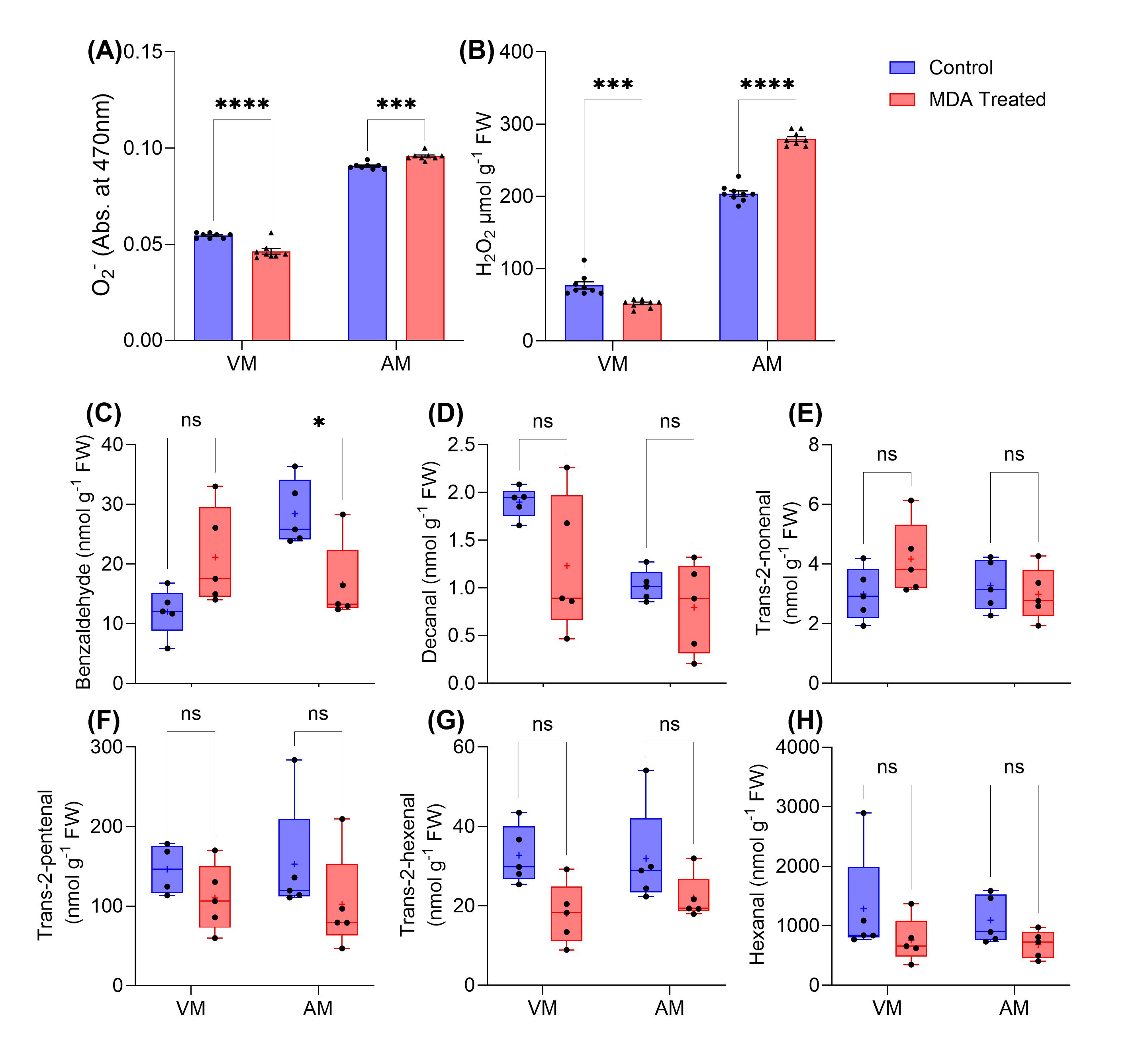

Supplement: Supplementary file 2 — Figure S2. Effect of exogenously applied 3 mM of malondialdehyde (MDA) on ROS and aldehyde levels in Sarcocornia fruticosa VM and Arthrocnemum macrostachyum (AM) 7 days after the application. (a) Level of superoxide accumulation 7 days after MDA application, (b) H2O2 content in plant shoot tips 7 days after MDA application. Data represent three biological replicates from three independent experiments (n = 9), and the error bar indicates standard error. Concentration of (c) benzaldehyde, (d) decanal, (e) trans‐2‐nonenal, (f) trans‐2‐pentenal, (g) trans‐2‐hexenal, and (h) hexanal in the control and 7 days after MDA application. Data represent five biological replicates randomly selected from three independent experiments (n = 5). Two‐way ANOVAs with Tukey's multiple comparison tests were used for identifying significant differences in values (ns = nonsignificant, *P‐value ≤0.05, ***P‐value ≤0.001, and ****P‐value ≤0.0001). [file TPJ-122-0-s001.jpg]

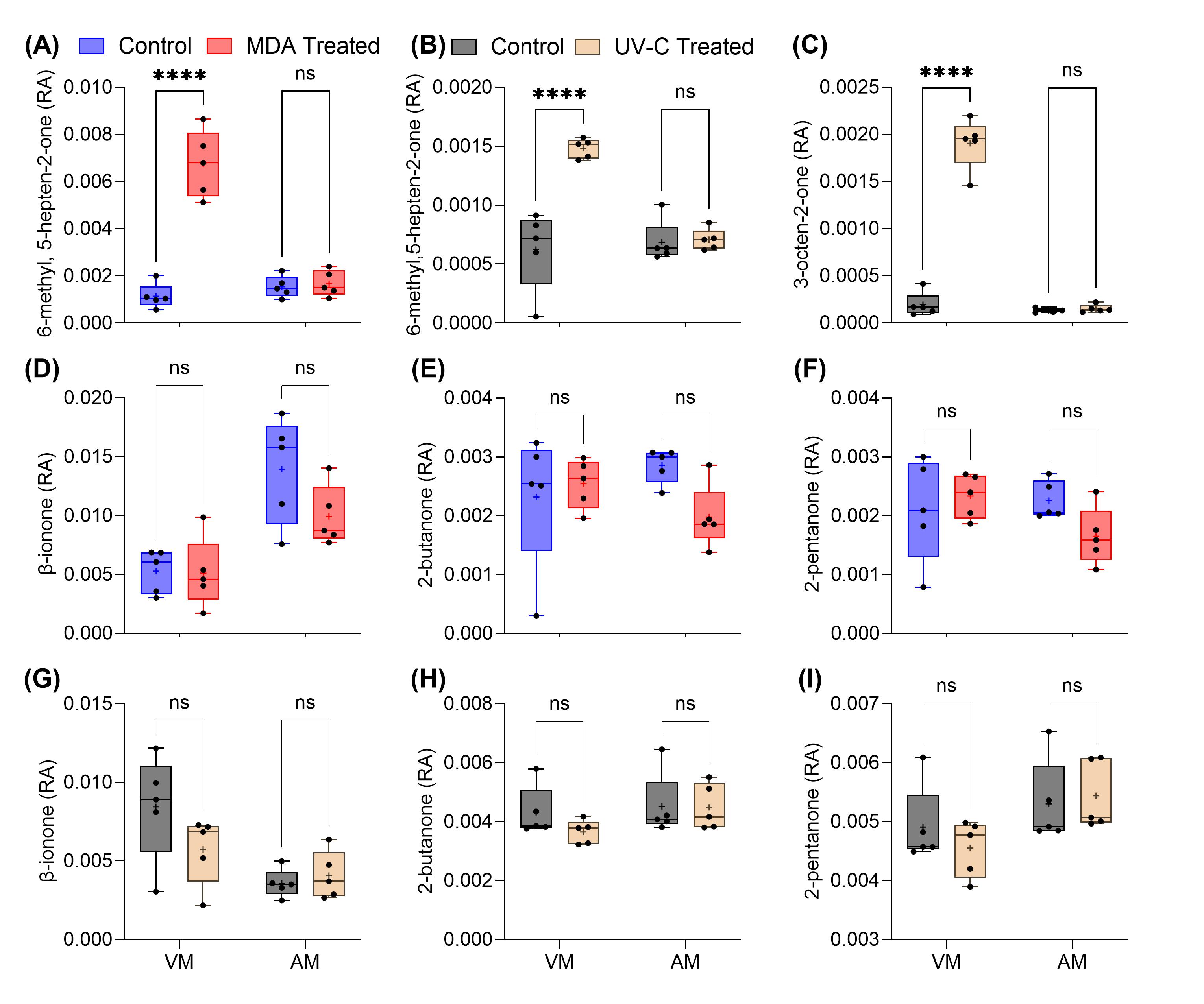

Supplement: Supplementary file 3 — Figure S3. Relative abundance (RA) of identified ketones in Sarcocornia fruticosa (VM) and Arthrocnemum macrostachyum (AM) exposed to UV‐C irradiation and MDA spray. (a) 6‐methyl, 5‐hepten‐2‐one in 3‐mM‐MDA‐treated plants. (b) 6‐methyl, 5‐hepten‐2‐one and (C) 3‐octen‐2‐one in UV‐C‐treated plants. (d) β‐ionone, (e) 2‐butanone, and (f) 2‐pentenone in MDA‐treated plants. (g) β‐ionone, (h) 2‐butanone, and (i) 2‐pentenone in UV‐C treated plants. Two‐way ANOVAs with Tukey's multiple comparison tests were used for identifying significant differences in values (ns = nonsignificant and ****P‐value ≤0.0001). [file TPJ-122-0-s003.jpg]
